# Prognosis and diastolic dysfunction predictors in patients with heart failure and recovered ejection fraction

**DOI:** 10.1038/s41598-022-12823-z

**Published:** 2022-05-24

**Authors:** Takuma Takada, Katsuhisa Matsuura, Yuichiro Minami, Takuro Abe, Ayano Yoshida, Makoto Kishihara, Shonosuke Watanabe, Shota Shirotani, Kentaro Jujo, Nobuhisa Hagiwara

**Affiliations:** 1 Department of Cardiology, Tokyo Women’s Medical University, 8-1 Kawadacho, Shinjuku-ku, Tokyo, 162-8666 Japan; 2 Institute of Advanced Biomedical Engineering and Science, Tokyo Women’s Medical University, Tokyo, Japan

**Keywords:** Cardiology, Interventional cardiology

## Abstract

There is limited data on whether diastolic dysfunction in patients with heart failure (HF) and recovered ejection fraction (HFrecEF) is associated with worse prognosis. We retrospectively assessed 96 patients diagnosed with HFrecEF and created ROC curve of their diastolic function at the 1-year follow-up for the composite endpoint of cardiovascular death and HF readmission after the follow-up. Eligible patients were divided into two groups according to the cutoff value of E/e′ ratio (12.1) with the highest AUC (0.70). Kaplan–Meier analysis showed that HFrecEF with high E/e′ group had a significantly poorer prognosis than the low E/e′ group (log-rank, *p* = 0.01). Multivariate Cox regression analysis revealed that the high E/e′ group was significantly related to the composite endpoint (hazard ratio 5.45, 95% confidence interval [CI] 1.23–24.1). The independent predictors at discharge for high E/e′ ratio at the 1-year follow-up were older age and female sex after adjustment for covariates (odds ratio [OR] 1.07, 95% CI 1.01–1.13 and OR 4.70, 95% CI 1.08–20.5). In conclusion, HFrecEF with high E/e′ ratio might be associated with a poor prognosis. Older age and female sex were independent predictors for a sustained high E/e′ ratio in patients with HFrecEF.

## Introduction

Improvement in systolic function—such as left ventricular ejection fraction (LVEF)—is sometimes experienced in patients with heart failure (HF) and reduced ejection fraction (HFrEF)^1^. Patients with HF and recovered ejection fraction (HFrecEF) demonstrate relatively better clinical outcomes than patients with persistent HFrEF^2–6^. It was previously reported that the independent predictors for improving LVEF were young age, female sex, and an etiology of non-ischemic heart disease^3,6,7^. Meanwhile, the withdrawal of guideline-directed medical therapy (GDMT) for HF in patients with dilated cardiomyopathy and recovered LVEF led to relapse of cardiomyopathy^8^. These results suggest that improvements in LVEF and recovery or remission of the injured myocardium may be different; therefore, patients with HFrecEF may be at risk of future cardiovascular (CV) events. It is, thus, important to clarify the subset of individuals with HFrecEF that may have a poor prognosis, despite exhibiting an improvement in systolic function.

Although, there is limited data regarding appropriate risk stratification and management in patients with HFrecEF, we hypothesized that diastolic function would be the prognostic indicator among HFrecEF patients as it is based on findings regarding the functional phenotypes of bioengineered cardiac tissue in hypoxia and reoxygenation. Both the systolic and relaxation functions of the cardiac tissue had deteriorated in the hypoxic condition, while in reoxygenation, the relaxation dysfunction remained, even after systolic function had fully recovered^9^. These results suggest that, in hypoxia, the relaxation dysfunction might be prolonged and the recovery or remission difficult to achieve when compared with systolic dysfunction. Relaxation dysfunction is one of the component of diastolic dysfunction^10^. Peak velocity of the early wave (E) to early diastole (e′) (E/e′) ratio and left atrial volume index (LAVI) assessed by echocardiography were correlated with LV filling pressure as well as the indexes for diagnosis of LV diastolic dysfunction^10,11^.

The aims of our study were to elucidate the predictors for sustained diastolic dysfunction at, as well as the long-term prognoses after, the 1-year follow-up.

## Methods

### Study population and endpoints

**Figure 1:**
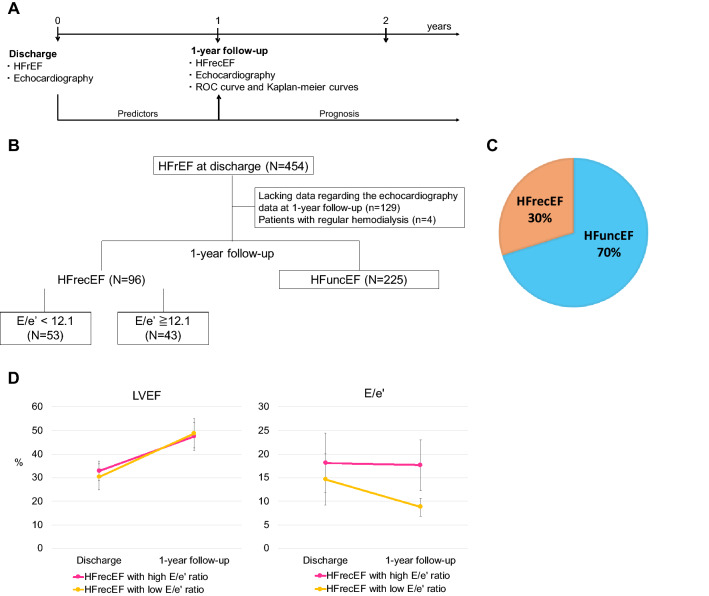
Study population. (**A**) Timeline. (**B**) Study flow chart. (**C**) Proportion of HFrecEF at the 1-year follow-up in patients with HFrEF. (**D**) Changes in LVEF and E/e′ ratio in HFrecEF with low or high E/e′ ratio group. *E/e′* peak velocity of the early wave (E) to early diastole (e′), *HF* heart failure, *HFrecEF* heart failure with recovered ejection fraction, *HFrEF* heart failure with reduced ejection fraction, *HFuncEF* heart failure with unchanged ejection fraction, *ROC* receiver operating characteristic.

We retrospectively assessed consecutive patients who were hospitalized for HFrEF and discharge alive at the Tokyo Women’s Medical University Hospital between July 2013 and October 2018. We followed-up and reassessed them via echocardiography at the 1-year follow-up and we confirmed that the patients were either diagnosed with HFrecEF or not. Inclusion criteria were follows: a diagnosis of HFrecEF at the 1-year follow-up; more than 1 year of follow-up; and obtaining the data describing the LVEF and E/e′ (septal e′) ratio (Fig. 1A,B).

Patients were diagnosed with HF using the Framingham HF diagnostic criteria^12^: HFrEF was defined as HF and an LVEF < 40% at discharge^13,14^; HFrecEF was defined as an LVEF < 40% at discharge that improved to ≥ 40% at the 1-year follow-up, and this was based on the results of previous studies^3,7,14^. Exclusion criteria were as follows: a diagnosis of heart failure with unchanged ejection fraction (HFuncEF: LVEF < 40% at discharge and 1-year follow-up)^7^; receipt of regular hemodialysis; and missing data describing the LVEF or peak velocity of the E/e′ ratio at the 1-year follow-up.

Next, we created the receiver operating characteristic (ROC) curve of their diastolic function at the 1-year follow-up for the composite endpoint of CV death and HF readmission after the follow-up. Eligible patients were divided into two groups according to the calculated cut-off value with highest area under the curve (AUC) among the four parameters related to the diastolic function: E/e′ ratio, LAVI, maximal tricuspid regurgitation velocity (TR Vmax), and e′^9^. CV death included death caused by acute myocardial infarction, sudden cardiac death, HF, stroke, CV procedures, CV hemorrhage, and other CV events^15^.

During the study period, 454 patients with HFrEF who were discharged alive, were followed up. Of those, four patients with regular hemodialysis and 129 patients with lacking data regarding the echocardiography data at 1-year follow-up were excluded. Of the remaining 321 patients, 225 patients (70%) did not exhibit improved LVEF at the 1-year follow-up and were diagnosed with HFuncEF. Finally, 96 patients (30%) with HFrecEF were analyzed (Fig. 1B,C). The study population was divided into two groups according to the cutoff value of the E/e′ ratio, as this exhibited the highest AUC among the four parameters. Fifty-three patients (55%) were classified into the low E/e′ ratio group, while 43 patients (45%) were classified into the high E/e′ ratio group (Fig. 1B).

The present study was approved by the Institutional Review Board of the Tokyo Women’s Medical University Ethical Committee (Approval Number: 2020–0028), conformed to the principles of the Declaration of Helsinki, and exempted from informed consent requirements owing to its retrospective design.

### Data collection

Patients’ clinical data at discharge were recorded, including vital signs, past medical history, oral medications, echocardiographic parameters, and laboratory data (complete blood count, estimated glomerular filtration rate [eGFR], and hemoglobin, total bilirubin, electrolyte, C-reactive protein, brain natriuretic peptide [BNP], and total cholesterol levels). Echocardiographic parameters—including the heart rhythm, left atrial diameter (LAD), LAVI, left ventricular end-diastolic diameter (LVDd), left ventricular end-systolic diameters (LVDs), LVEF, E/e′, e′, deceleration time, TR Vmax, estimated right ventricular systolic pressure (RVSP), maximum and minimum inferior vena cava (IVC) diameter, left ventricular mass index (LVMI), and the presence of atrial arrhythmias at echocardiography—were evaluated at discharge and the 1-year follow-up. Transthoracic echocardiography was performed using a Vivid 7 (GE Healthcare, Horten, Norway) or iE33 (Philips Healthcare, Bothell, WA, USA) ultrasound system. LVDd, LVDs, and LAD were recorded in the parasternal long-axis view, and the LVEF and LAVI were calculated using the modified Simpson method. The estimated RVSP was calculated from the TR Vmax: estimated RVSP = 4 × (TR Vmax)^2^ + right atrial pressure. Atrial arrhythmias were defined as the heart rhythm of atrial fibrillation, atrial flutter, or atrial tachycardia^16,17^. The eGFR was calculated using previously published equations: eGFR (mL/min/1.73 m^2^) = 194 × serum creatinine^(−1.094)^ × age^(−0.287)^ (× 0.739, if female)^18^. Anemia was defined as hemoglobin levels < 12.0 g/dL in women and < 13.0 g/dL in men^13^. The definition of ischemic cardiomyopathy was that left ventricular (LV) dysfunction due to severe ischemic heart disease including the myocardial infarction and angina pectoris according to Japanese Circulation Society 2018 guideline on diagnosis and treatment of cardiomyopathies.

### Statistical analyses

The data are expressed as means and standard deviations, median values and interquartile range (IQR), or percentages, as appropriate. The Student’s t-test was used to compare normally distributed continuous variables between the groups, while the Mann–Whitney U test was used for skewed continuous variables; Fischer’s exact test was used to evaluate the categorical variables. A paired t-test was used to compare the LVEF or E/e′ ratio at discharge and the 1-year follow-up. Pearson product-moment correlation coefficient was used to identify the correlation between E/e′ ratio at the 1-year follow-up and LVEF changes from the discharge to the 1-year follow-up. We plotted ROC curves for the composite of CV death and readmission for HF using the E/e′, LAVI, TR Vmax, and e′ at the 1-year follow-up; additionally, we estimated the optimal cutoff value based on the Youden index. BNP levels were log-transformed. Logistic regression analysis was performed to identify independent factors at discharge related to high E/e′ ratio at the 1-year follow-up. Variables were considered clinically significant if they reached a significance level of *p* < 0.05 and were subsequently included in the multivariable logistic regression model. The Kaplan–Meier method and log-rank tests were used to compare the event-free survival ratios between the two groups during the follow-up period after the 1-year follow-up. Multivariable Cox regression analysis was performed to assess whether high E/e′ ratio at the 1-year follow-up was associated with the primary endpoint after adjusting for covariates. Due to small number of patients who experienced the composite endpoint, the multivariate analysis was performed by adjusting for age and sex only. Two-sided significance was set at *p* < 0.05. Furthermore, sensitivity analysis was performed to remove the influence of different definition of HFrecEF on the results. We applied another definition of HFrecEF for the sensitivity analysis. The definition was (1) decreased LVEF < 40% at baseline; (2) ≥ 10% absolute improvement in LVEF; and (3) a second measurement of LVEF ≥ 40%, according to the previous reports^19^. All statistical analyses were performed using R software (version 1.41.1; R Foundation for Statistical Computing, Vienna, Austria)^20^.

## Results

The median follow-up duration was 537 (IQR 309–923) days after the 1-year follow-up. During the study period, 14 patients (15%) were readmitted for HF or died as a consequence of CV events; only one patient died from CV disease.

**Figure 2:**
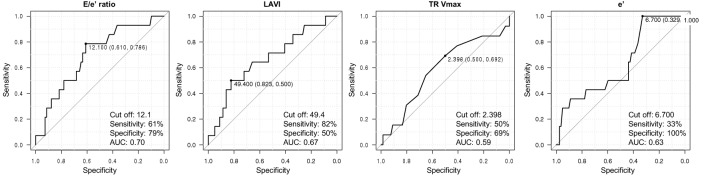
ROC curve of E/e′ ratio, LAVI, TR Vmax, and e′ at the 1-year follow-up for CV death and readmission for HF after the 1-year follow-up in patients with HFrecEF. *CV* cardiovascular, *E/e′* peak velocity of the early wave (E) to early diastole (e′), *HF* heart failure, *HFrecEF* heart failure with recovered ejection fraction, *LAVI* left atrial volume index, *ROC* receiver operating characteristic, *TR Vmax* maximal tricuspid regurgitation velocity.

The ROC curve for a composite of CV death and readmission of HF using the E/e′ ratio, LAVI, TR Vmax, and e′ at 1-year follow-up revealed cutoff values of 12.1, 49.4 mL/m^2^, 2.40 m/s, and 6.7 cm/s, respectively (AUC 0.70, 95% confidence interval [CI] 0.55–0.84; AUC 0.67, 95% CI 0.50–0.83; AUC 0.59, 95% CI 0.41–0.77; AUC 0.63, 95% CI 0.48–0.79, respectively; Fig. 2). There were no statistically significant differences regarding the AUC between the E/e′ ratio and LAVI at the 1-year follow-up (*p* = 0.76).

### Patient characteristics at discharge

Table 1 shows the patient characteristics of the study population and comorbidities at discharge. Significant differences were evident between the two groups concerning age, sex, principal cause of HF due to ischemic cardiomyopathy, and sodium, hemoglobin, and BNP levels. Regarding echocardiography during the hospitalization, LVEF and E/e′ were higher in the HFrecEF with high E/e′ group than the HFrecEF with low E/e′ group. The estimated RVSP, E-wave deceleration time, e′, LAVI, LVMI, IVC diameter, and presence of atrial arrhythmias at the echocardiography were comparable between the two groups. Angiotensin-converting enzyme inhibitors or angiotensin receptor blocker inhibitors (ACEi/ARB) and beta-blockers were prescribed in approximately 90% of patients at discharge; statin was more often prescribed in the high E/e′ than low E/e′ group.

**Table 1:** Patients’ characteristics at discharge according to HFrecEF with low or high E/e′ ratio at the 1-year follow-up.

Variables	All patients	HFrecEF with low E/e′ (< 12.1)	HFrecEF with high E/e′ (≥ 12.1)	*p *value
	n = 96	n = 53	n = 43	
Age, year	59 ± 15	51 ± 14	68 ± 11	< 0.001
Female	29 (30%)	9 (17%)	20 (47%)	0.003
BMI, kg/m^2^	24 ± 5.1	24 ± 4.8	23 ± 5.4	0.06
HT	66 (69%)	35 (66%)	31 (72%)	0.66
Diabetes	40 (42%)	19 (36%)	21 (49%)	0.22
IDDM	7 (7%)	4 (8%)	3 (7%)	> 0.99
Dyslipidemia	56 (58%)	29 (55%)	27 (63%)	0.53
Smoking	50 (52%)	29 (55%)	21 (49%)	0.68
Family history of heart disease	23 (24%)	13 (25%)	10 (23%)	> 0.99
Atrial arrhythmias	39 (41%)	20 (38%)	19 (44%)	0.54
Prior CABG	2 (2%)	1 (2%)	1 (2%)	> 0.99
Prior myocardial infarction	8 (8%)	2 (4%)	6 (14%)	0.13
Prior stroke	3 (3%)	2 (4%)	1 (2%)	> 0.99
PM	11 (11%)	3 (6%)	8 (19%)	0.06
ICD	2 (2%)	0 (0%)	2 (5%)	0.20
CRT	0 (0%)	0 (0%)	0 (0%)	NS
Ischemic cardiomyopathy	18 (19%)	5 (9%)	13 (30%)	0.02
Systolic BP, mmHg	116 ± 18	114 ± 19	118 ± 16	0.28
Diastolic BP, mmHg	64 ± 12	66 ± 12	63 ± 12	0.19
Heart rate, bpm	72 ± 12	72 ± 12	71 ± 11	0.87
**Echocardiography**				
LVEF, %	31 ± 5.2	30 ± 5.5	33 ± 4.1	0.01
LVDd, mm	59 ± 8.7	62 ± 8.2	57 ± 8.6	0.004
LVDs, mm	51 ± 8.9	54 ± 8.2	47 ± 8.6	< 0.001
LAD, mm	45 ± 7.6	46 ± 7.8	45 ± 7.4	0.76
RVSP, mmHg	36 ± 11	35 ± 9.1	38 ± 13	0.10
TR Vmax, m/s	2.5 ± 0.5	2.4 ± 0.5	2.6 ± 0.6	0.14
E-wave deceleration time, ms	153 [121–207]	142 [118–198]	161 [127–219]	0.16
E/e′	16 ± 6.0	15 ± 5.4	18 ± 6.3	0.005
e′, cm/s	5.1 ± 1.7	5.3 ± 1.8	4.7 ± 1.6	0.10
LAVI, mL	54 ± 24	52 ± 22	57 ± 27	0.32
LVMI, g/m^2^	129 ± 44	133 ± 49	124 ± 38	0.36
Maximum IVC diameter, mm	16 ± 5.0	16 ± 5.1	15 ± 4.9	0.42
Minimum IVC diameter, mm	9.2 ± 5.3	9.6 ± 5.6	8.7 ± 1.0	0.39
Aortic valve regurgitation (moderate or severe)	4 (4%)	2 (4%)	2 (5%)	> 0.99
Mitral valve regurgitation (moderate or severe)	20 (21%)	11 (21%)	9 (21%)	> 0.99
Atrial arrhythmias at echocardiography	26 (27%)	11 (21%)	15 (35%)	0.17
**Lab data**				
WBC, /μL	5680 [4615–6803]	5340 [4550–6700]	5990 [5060–7075]	0.18
Hemoglobin, g/dL	14 ± 2.2	14 ± 2.0	13 ± 2.2	0.004
Anemia	31 (32%)	15 (28%)	16 (37%)	0.39
Total bilirubin, mg/dL	0.8 [0.6–1.0]	0.8 [0.6–1.1]	0.7 [0.6–1.0]	0.57
eGFR, mL/min/1.73m^2^	50 ± 27	51 ± 24	46 ± 28	0.27
Sodium, mEq/L	139 [139–141]	140 [139–142]	139 [138–141]	0.03
Potassium, mEq/L	4.4 ± 0.5	4.4 ± 0.4	4.4 ± 0.6	0.98
Chloride, mEq/L	103 ± 2.9	103 ± 2.2	103 ± 3.5	0.27
T-Chol, mg/dL	176 ± 37	178 ± 41	175 ± 33	0.69
CRP, mg/dL	0.2 [0.1–0.7]	0.2 [0.1–0.6]	0.2 [0.1–0.7]	0.64
BNP, pg/mL	180 [78–360]	114 [59–313]	262 [128–470]	0.005
**Medication at discharge**				
ACEi/ARB	85 (89%)	46 (87%)	39 (91%)	0.75
Beta blocker	92 (96%)	51 (96%)	41 (95%)	> 0.99
Aldosterone antagonist	69 (72%)	40 (75%)	29 (67%)	0.49
Thiazide	4 (4%)	1 (2%)	3 (7%)	0.32
Furosemide dose, mg/day	20 [20–40]	20 [10–40]	20 [20–40]	0.08
Calcium channel blocker	20 (21%)	10 (19%)	10 (23%)	0.62
Inotropes	9 (9%)	7 (13%)	2 (5%)	0.18
Statin	37 (39%)	15 (28%)	22 (51%)	0.03
Amiodarone	18 (19%)	12 (23%)	6 (14%)	0.31
OAC	42 (44%)	23 (43%)	19 (44%)	> 0.99
DPP4i	20 (21%)	11 (21%)	9 (21%)	> 0.99
SGLT2i	3 (3%)	2 (4%)	1 (2%)	> 0.99

*ACEi* angiotensin converting enzyme inhibitor, *ARB* angiotensin receptor blocker, *OAC* oral anti-coagulants, *BMI* body mass index, *BNP* brain natriuretic peptide, *BP* blood pressure, *bpm* beats per minute, *CABG* coronary artery bypass grafting, *CRP* C-reactive protein, *CRT* cardiac resynchronization therapy, *DPP4i* dipeptidyl peptidase-4 inhibitor, *E/e′* peak velocity of the early wave (E) to early diastole (e′) ratio, *eGFR* estimated glomerular filtration rate, *HDL* high density lipoprotein, *HFrecEF* heart failure with recovered ejection fraction, *HT* hypertension, *ICD* implantable cardioverter defibrillator, *IDDM* insulin dependent diabetes mellitus, *IVC* inferior vena cava, *LAD* left atrial dimension, *LAVI* left atrial volume index*, LDL* low density lipoprotein, *LVEF* left ventricular ejection fraction, *LVDd* left ventricular end-diastolic diameter, *LVDs* left ventricular end-systolic diameter, *LVMI* left ventricular mass index, *OAC* oral anticoagulants, *PM* pacemaker, *RVSP* right ventricular systolic pressure, *SGLT2i* sodium-glucose cotransporter 2 inhibitor, *T-Chol* total cholesterol, *TR* *Vmax* maximal tricuspid regurgitation velocity, *WBC* white blood cell.

### Patient characteristics and echocardiography data at the 1-year follow-up

The median follow-up period between discharge and the 1-year follow-up was 365 (IQR 332–398) days. Although LVEF in both HFrecEF with low and high E/e′ ratio groups significantly improved from 30 ± 5.5 and 33 ± 4.1% at discharge to 49 ± 6.1% and 47 ± 5.8% at the 1-year (paired-t test: *p* < 0.001, respectively, Fig. 1D), E/e′ ratio in HFrecEF with high E/e′ group were unchanged from 18 ± 6.3 at discharge to 18 ± 5.4 at the 1-year follow-up (paired-t test: *p* = 0.68), while E/e′ ratio in HFrecEF with low E/e′ group improved from 15 ± 5.4 at discharge to 8.8 ± 1.9 at the 1-year follow-up (paired-t test: *p* < 0.001, Fig. 1D). Interestingly, there was inversely mild correlation between the E/e′ ratio at the 1-year follow-up and LVEF changes (%) from the discharge to the 1-year follow-up (*r* = − 0.25,* p* = 0.02, Supplemental Fig. 1). There were no significant differences in systolic blood pressure, diastolic blood pressure, heart rate, atrial arrhythmias at the 1-year follow-up, LVEF, LVDd, LVDs, LVMI, E-wave deceleration time, IVC diameter, the presence of atrial arrhythmias at the echocardiography, and the prescription rates of GDMT for HF between the two groups at the 1-year follow-up. Changes in heart rate from discharge to the 1-year follow-up did not differ between the two groups (low E/e′ group: − 2.4 ± 14 bpm vs. high E/e′ group: 0.8 ± 18 bpm; *p* = 0.33). Patients in the high E/e′ group had larger LADs and LAVIs, as well as a tendency to exhibit a higher RVSP and TR Vmax, compared with those in the low E/e′ group. Then, e′ was lower and BNP level was higher in HFrecEF with high E/e′ group compared with HFrecEF with low E/e′ group. There was statistically significant difference in the furosemide dose between the two groups. No patient had moderate to severe mitral valve regurgitation. The prescription rates for ACEi/ARB and β-blockers were compatible between discharge and the 1-year follow-up; however, that of aldosterone antagonists decreased from 72% at discharge to 58% at the 1-year follow-up (Table 2).

**Table 2:** Patients’ characteristics and echocardiography data at the 1-year follow-up.

Variables	All patients	HFrecEF with low E/e′ (< 12.1)	HFrecEF with high E/e′ (≥ 12.1)	*p *value
	n = 96	n = 53	n = 43	
Systolic BP, mmHg	121 ± 18	118 ± 15	124 ± 21	0.11
Diastolic BP, mmHg	72 ± 13	71 ± 11	73 ± 15	0.32
Heart rate, bpm	69 ± 12	68 ± 11	71 ± 13	0.31
Atrial arrhythmias (comorbidity)	41 (43%)	21 (40%)	20 (47%)	0.54
**Echocardiography**				
LVEF, %	48 ± 6.0	49 ± 6.1	47 ± 5.8	0.32
LVDd, mm	51 ± 6.4	52 ± 6.2	51 ± 6.6	0.50
LVDs, mm	39 ± 5.8	39 ± 5.7	39 ± 6.0	0.93
LAD, mm	40 ± 7.5	39 ± 7.5	42 ± 7.0	0.009
RVSP, mmHg	34 ± 7.4	32 ± 7.2	35 ± 7.6	0.08
TR Vmax, m/s	2.4 ± 0.4	2.3 ± 0.3	2.5 ± 0.4	0.07
E-wave deceleration time, ms	201 ± 49	203 ± 39	200 ± 59	0.81
E/e′	13 ± 5.9	8.8 ± 1.9	18 ± 5.4	< 0.001
e′, cm/s	6.0 ± 2.6	7.1 ± 2.9	4.6 ± 1.2	< 0.001
LAVI, mL/m^2^	34 [28–46]	32 [25–38]	42 [33–53]	0.002
LVMI, g/m^2^	94 [74–110]	89 [74–99]	97 [76–113]	0.23
Maximum IVC diameter, mm	12 ± 4.1	12 ± 4.1	12 ± 4.2	0.81
Minimum IVC diameter, mm	6.4 ± 3.1	6.2 ± 2.8	6.6 ± 3.5	0.49
Aortic valve regurgitation (moderate or severe)	1 (1**%**)	1 (2**%**)	0 (0**%**)	> 0.99
Mitral valve regurgitation (moderate or severe)	0 (0**%**)	0 (0%)	0 (0%)	NS
Atrial arrhythmias at echocardiography	14 (15%)	6 (11%)	8 (19%)	0.39
BNP, pg/mL	54 [17–132]	21 [8–56]	111 [56–296]	< 0.001
ACEi/ARB	85 (89%)	47 (89%)	38 (88%)	> 0.99
Beta blocker	93 (97%)	52 (98%)	41 (95%)	0.59
Aldosterone antagonist	56 (58%)	35 (66%)	21 (49%)	0.10
Furosemide dose, mg/day	18 [0–20]	10 [0–20]	20 [10–40]	< 0.001

*ACEi* angiotensin converting enzyme inhibitor, *ARB* angiotensin receptor blocker, *BNP* brain natriuretic peptide, *BP* blood pressure, *bpm* beats per minute, *E/e′ *peak velocity of the early wave (E) to early diastole (e′) ratio, *HFrecEF* heart failure with recovered ejection fraction, *IVC* inferior vena cava, *LAD* left atrial dimension, *LAVI* left atrial volume index, *LVEF* left ventricular ejection fraction, *LVDd* left ventricular end-diastolic diameter, *LVDs* left ventricular end-systolic diameter, *LVMI* left ventricular mass index, *RVSP* right ventricular systolic pressure, *TR* *Vmax* maximal tricuspid regurgitation velocity.

### Prognosis

**Figure 3:**
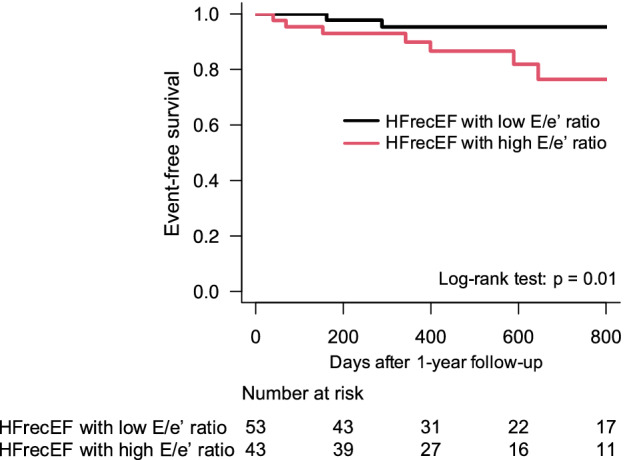
Composite outcome after the 1-year follow-up. Kaplan–Meier curve of the composite outcome between the two groups categorized according to the E/e′ ratio at the 1-year follow-up using a cutoff score of 12.1. E/e′ = peak velocity of the early wave (E) to early diastole (e′).

The Kaplan–Meier curve revealed that the rate of the composite endpoint was significantly higher in the high E/e′ group than the low E/e′ group (log-rank test, *p* = 0.01; Fig. 3). Furthermore, multivariate Cox regression analysis revealed that an E/e′ ratio ≥ 12.1 at the 1-year follow-up was associated with the composite endpoint after the 1-year follow-up, after adjusting for age and sex (hazard ratio [HR] 5.45, 95% CI 1.23–24.1; Table 3). This result was consistent with sensitivity analysis with other definition of HFrecEF (Supplemental Fig. 2).

**Table 3:** Cox regression analysis for the composite of CV death and heart failure readmission after the 1-year follow-up.

	Model type	Hazard ratio	95%CI	*p *value
HFrecEF with low E/e′ (< 12.1)	Reference	1.00		
HFrecEF with high E/e′ (≥ 12.1)	Univariate	4.51	1.23–16.2	0.02
	Age and sex adjusted	5.45	1.23–24.1	0.03

*CI* confidence interval, *CV* cardiovascular, *E/e*′  peak velocity of the early wave (E) to early diastole (e′) ratio, *HFrecEF* heart failure with recovered ejection fraction.

Meanwhile, when the study population was divided into two groups according to a cutoff of 49.4 mL/m^2^ for LAVI, the Kaplan–Meier curve demonstrated that patients in the high LAVI group exhibited higher rate of the composite endpoint, compared with those in the low LAVI group (log-rank test, *p* *= *0.02; Supplemental Fig. 3). However, the age and sex adjusted HR for the composite endpoint did not significantly differ between the two groups (HR 1.86, 95% CI 0.60–5.73).

### Predicting high E/e′ ratio at the 1-year follow-up

Discharge parameters including age, female sex, ischemic cardiomyopathy, high BNP level, low sodium level, high LVEF, high E/e′ ratio, and a prescription for statins correlated with a high E/e′ ratio at the 1-year follow-up in the univariate logistic regression analyses. After adjusting for these parameters, older age and female sex remained independent predictors for high E/e′ ratio at the 1-year follow-up (odds ratio [OR] 1.07, 95% CI 1.01–1.13 and OR 4.70, 95% CI 1.08–20.5, respectively; Table 4).

**Table 4:** Predictors of HFrecEF with high E/e′ ratio at the 1-year follow-up using the comorbidities and patients’ characteristics at discharge.

Patients’ characteristics at discharge	Univariate			Multivariate		
	OR	95% CI	*p *value	OR	95% CI	*p *value
Age, per 1 year	1.11	1.06–1.16	< 0.001	1.07	1.01–1.13	0.02
Female	4.25	1.67–10.8	0.002	4.70	1.08–20.5	0.04
BMI, per 1 kg/m^2^	0.93	0.85–1.01	0.07			
Hypertension	1.33	0.55–3.19	0.53			
Diabetes mellitus	1.71	0.75–3.88	0.20			
Prior CABG	1.24	0.07–20.4	0.88			
Family history of heart disease	0.93	0.36–2.40	0.89			
Atrial arrhythmias	1.31	0.58–2.96	0.52			
Ischemic cardiomyopathy	4.16	1.35–12.8	0.01	3.23	0.45–24.1	0.24
Systolic BP, per 1 mmHg	1.01	0.99–1.04	0.28			
Heart rate, per 1 bpm	1.00	0.96–1.03	0.87			
Log-transferred BNP, per 10 pg/mL	3.70	1.36–10.0	0.01	2.55	0.65–9.96	0.18
eGFR, per 1 mL/min/1.73m^2^	0.99	0.98–1.01	0.38			
Anemia	1.50	0.64–3.55	0.35			
Total bilirubin, per 1 mg/dL	0.81	0.32–2.10	0.67			
CRP, per 1 mg/dL	1.26	0.82–1.92	0.29			
Sodium, per 1 mEq/L	0.76	0.61–0.93	0.009	0.80	0.57–1.14	0.22
LVEF, per 1%	1.12	1.02–1.22	0.01	1.09	0.94–1.26	0.26
LAD, per 1 mm	1.01	0.96–1.06	0.76			
RVSP, per 1 mmHg	1.03	0.99–1.08	0.11			
TR Vmax, per 1 m/s	1.90	0.81–4.45	0.14			
E-wave deceleration time, per 1 ms	1.00	0.99–1.01	0.64			
E/e′, per 1	1.10	1.03–1.19	0.007	1.05	0.93–1.17	0.44
e′, per 1 cm/s	0.81	0.62–1.05	0.11			
LAVI, per 1 mL/m^2^	1.01	0.99–1.03	0.32			
LVMI, per 1 g/m^2^	1.00	0.96–1.01	0.36			
ACEi/ARB	1.48	0.40–5.45	0.55			
Beta blocker	0.80	0.11–5.96	0.83			
Calcium channel blocker	1.30	0.49–3.50	0.60			
Aldosterone antagonist	0.67	0.28–1.65	0.39			
Furosemide daily dose, per 1 mg/day	1.02	0.99–1.05	0.09			
Statin	2.65	1.14–6.18	0.02	4.11	0.96–17.7	0.06

*ACEi* angiotensin converting enzyme inhibitor, *ARB* angiotensin receptor blocker, *BMI* body mass index, *BNP* brain natriuretic peptide, *BP* blood pressure, *bpm* beats per minute, *CABG* coronary artery bypass grafting, *CI* confidence interval, *CRP* C-reactive protein, *E/e′ *peak velocity of the early wave (E) to early diastole (e′) ratio, *eGFR* estimated glomerular filtration rate, *LAD* left atrial dimension, *LAVI* left atrial volume index, *LVEF* left ventricular ejection fraction, *LVMI* left ventricular mass index, *OR* odds ratio, *RVSP* right ventricular systolic pressure, *TR* *Vmax* maximal tricuspid regurgitation velocity.

## Discussion

This study investigated the predictors at discharge for high E/e′ ratio at the 1-year follow-up in patients with HFrecEF, as well as their prognosis. The principal findings were as follows: (1) patients with HFrecEF and high E/e′ ratio at the 1-year follow-up had a poor prognosis after the 1-year follow-up, and (2) older age and female sex at the hospitalization were associated with a high E/e′ ratio at the 1-year follow-up.

Several studies have been conducted regarding the recovery of LVEF during the follow-up period^2–6^. The current study reveals that the proportion of HFrecEF at the 1-year follow-up in patients with HFrEF is consistent with previous reports^3,4^. Additionally, Bermejo et al. reported that the percentage of the implantable cardioverter defibrillator (ICD) implantation, ischemic etiology of HF, ACEi/ARB, and beta blocker in patients with HFrecEF as the predictors of LVEF improvement were 3%, 20%, 90%, and 78%, respectively^21^. Those percentages were similar to those of the current study; ICD implantation: 2%, ischemic etiology of HF: 19%, ACEi/ARB: 89%, and beta blocker: 96% (Table 1). However, despite the LVEF improving in patients with HFrEF, the predictors of remaining diastolic dysfunction and the relationship between prognosis and diastolic function at follow-up remain unknown. We thus focused on patients with HFrecEF and their diastolic function at follow-up. We selected the E/e′ ratio at the 1-year follow-up to classify the study population as the diastolic dysfunction parameter because this parameter demonstrated the highest AUC for the composite endpoint when compared with LAVI, e′, or TR Vmax, which were also related to the diastolic dysfunction. The E/e′ ratio is one of the parameters assessing left ventricular filling pressures, which can be assessed by combining the effects of the transmitral driving pressure and myocardial relaxation^22^. Patients in HFrecEF with high E/e′ ratio group had lower e′ (< 7 cm/s) compared with patients in HFrecEF with low E/e′ group, suggesting that patients in HFrecEF with high E/e′ ratio group had the relaxation dysfunction at the 1-year follow-up^10,11^. Furthermore, patients in the high E/e′ group had a larger LAVI (> 34 mL/mm^2^) and higher TR Vmax than those in the low E/e′ group at the 1-year follow-up. It suggested that patients in the high E/e′ group exhibited diastolic dysfunction regardless of the improvement in LVEF, in reference to previous reports^10,23^. Table 1 showed that HFrecEF with high E/e′ group had higher LVEF and smaller LV size than HFrecEF with low E/e′ group. HFrecEF with high E/e′ group had higher proportion of female patients compared with low E/e′ group, which might influence the LV size. Further, sex-specific differences in the distribution of LVEF was reported in nationwide register (N = 499,153), which demonstrated that mean LVEF was higher in women than men^24^. We speculated that sex differences between the two groups might affect the LVEF and LV size at the discharge. In addition, because Zhang et al. reported that the degree of LVEF improvement was associated with the prognosis^25^, we created ROC curve and compared the AUC of E/e′ ratio at the 1-year follow-up and LVEF changes from the discharge to the 1-year follow-up for the composite of CV death and rehospitalization for HF. Although there was no significant difference between them, AUC was higher in E/e′ ratio than LVEF change (E/e′ ratio: 0.70 vs. LVEF change: 0.61, *p* = 0.48, Supplemental Fig. 4).

In previous studies, patients with HFrecEF were shown to have a lower risk of mortality or hospitalization for HF^3–5^. One of the predictors of increased EF was female sex^3,6,7^. Interestingly, elderly women in the current study tended to exhibit diastolic dysfunction at the 1-year follow-up, regardless of recovered LVEF. This can be explained by the effect of sex hormones as postmenopausal women with low levels of estrogen are prone to cardiac diastolic dysfunction through the suppression of sarcoplasmic reticulum Ca^2+^-ATPase (SERCA) activity^26,27^. Furthermore, menopause is associated with a reduction in cGMP-protein kinase G signaling by decreasing plasma natriuretic peptide levels^28–30^. These mechanisms may lead to CV events. Although Ghimire et al. reported that female patients exhibited lower mortality risk than men among HFrecEF patients^31^, the impact of sex differences on the prognosis was not evaluated in the current study because of small sample size. HFrecEF with high E/e′ ratio could indicate a more severe comorbidity, potentially causing the poor prognosis.

Regarding the possible reason for persistent diastolic dysfunction, the fabricated human cardiac tissue revealed systolic and relaxation dysfunction in a hypoxic environment^9^. After reoxidation, systolic function had completely improved; however, the relaxation dysfunction remained, with the mRNA expression of phospholamban upregulated. This suggests that compared with systolic dysfunction, it might be difficult to achieve normalization of the relaxation dysfunction in hypoxia. The myocardium in heart failure is constantly exposed to the hypoxic and normoxemic environment. In cases of acute decompensated HF, the myocardium may be exposed to the hypoxic environment as a result of decreasing oxygen supply due to pulmonary congestion—as well as increasing oxygen demand due to volume or pressure overload—regardless of the etiology of HF^32,33^. The microvascular density decreased in patients with chronic HF compared with healthy subjects^32,34,35^. Arnold et al. reported that the microvascular dysfunction in HF with preserved ejection fraction (HFpEF) was correlated with E/e′ ratio, which was related to the long-term prognosis^36^. On the other hand, the compensatory mechanisms in HF help to supply the oxygen and to suppress the oxygen demand as well as GDMT can improve the hypoxic condition in the myocardium by supporting the decrease of oxygen demand and suppression of sympathetic activity, thus possibly contributing to the improvement of systolic dysfunction^33,37^. Meanwhile, diastolic function was affected by the relaxation function, left atrial size, and fibrosis in the cardiac tissue, in addition to oxygen demand^38^. Therefore, as shown in a previous report^9^, recovery of diastolic dysfunction may be difficult in patients with HFrecEF who exhibit cardiac fibrosis due to aging, despite the condition being improved by GDMT.

As the clinical implication, patients with HFrecEF and high E/e′ ratio exhibited unfavorable clinical outcomes in this study. It was reported that the changes in heart rate during the follow-up periods were associated with relapse in patients with dilated cardiomyopathy and recovered LVEF^39^. However, heart rate at discharge and at the 1-year follow-up, as well as changes in heart rate, were compatible between the two groups in the current study; therefore, monitoring of the E/e′ ratio may be a useful risk stratification tool for future CV events in patients with HFrecEF. Meanwhile, Pritchett et al. reported that LAVI was also correlated with the diastolic dysfunction as well as long-term prognosis in a cross-sectional sample with > 45 years of age^40^. In the current study, AUC of E/e′ ratio at the 1-year follow-up for the composite endpoint was higher than that of LAVI (0.70 vs. 0.67), but not statistically significance between them (*p* = 0.76). It is still unclear which parameters are better for assessing the prognosis in patients with HFrecEF. The combination of the parameters might predict the prognosis accurately.

Although appropriate strategies for improving a patient’s diastolic dysfunction remain unestablished, and careful observation and management are needed for these patients, it was reported that the angiotensin receptor neprilysin inhibitor (ARNI) might be effective in female patients with HFpEF, those who are older (> 65 years; postmenopausal women), or those with relatively lower LVEF (< 57%)^31,41^. Furthermore, ARNI altered the biomarker of abnormal extracellular matrix (ECM) homeostasis and improved clinical outcomes in patients with HFpEF, likely through antifibrotic effects^42^. This may indicate that ARNI had favorable effects on diastolic dysfunction. In this study population, ARNI might be effective in patients with HFrecEF and high E/e′ ratio because of the mean age of 68 years, high rate of females, and mean LVEF of 47% at the 1-year follow-up. Therefore, we considered that clinicians should assess the implications of ARNI for patients with HFrecEF and diastolic dysfunction, which may lead to better clinical outcomes.

There were several limitations in this study. This was a retrospective study performed at a single center with a small sample size. There may be a selection bias as some patients were excluded from this study due to missing echocardiographic data. Additionally, patients who underwent mitral valve plasty or replacement were excluded as their E/e′ ratio could not be accurately measured. Although we grouped patients according to E/e′ ratio using a cutoff value of 12.1 (Youden index), other cutoff values—such as an E/e′ ratio of 14 or 15—were not assessed, since the number of patients with an E/e′ ratio greater than 14 was very small. Although we assessed the septal e′, the diagnostic accuracy of tissue Doppler for evaluating LV filling pressure and diastolic dysfunction have been still discussed^43^. We did not show the NYHA classification at the discharge and the 1-year follow-up because there was not enough amount of data on it. No patients were prescribed ARNI and ivabradine at both discharge and the 1-year follow-up as these drugs were not approved in Japan at the time, and sodium-glucose cotransporter 2 inhibitors were only approved for patients with diabetes during the study period; these differences may have influenced the results. The multivariate analysis might be overfitting. Further large-scale, prospective investigations are needed to determine the best management for patients with HFrecEF and high E/e′ ratio.

## Conclusions

Elderly and female patients hospitalized for HFrEF may exhibit diastolic dysfunction at the 1-year follow-up even if their LVEF had improved; additionally, patients with HFrecEF and high E/e′ ratio at the 1-year follow-up had a poor prognosis. Close observation and novel strategies are, thus, needed for this population.

## Supplementary Information

Supplementary Information.

## Data Availability

Data are available from the corresponding authors upon reasonable request.

## Abbreviations

BNP: Brain natriuretic peptide
CI: Confidence interval
CV: Cardiovascular
E/e′: Peak velocity of the early wave (E) to early diastole (e′)
HF: Heart failure
HFrecEF: Heart failure with recovered ejection fraction
HFrEF: Heart failure with reduced ejection fraction
LAVI: Left atrial volume index
LVEF: Left ventricular ejection fraction
TR Vmax: Maximal tricuspid regurgitation velocity
