# Supplementary material for: Prognosis and diastolic dysfunction predictors in patients with heart failure and recovered ejection fraction
Source: Sci Rep. 2022 May 24;12:8768. doi: 10.1038/s41598-022-12823-z (PMC9130289; doi:10.1038/s41598-022-12823-z)
Supplement: Supplementary file 1 — Supplementary Information. [file 41598_2022_12823_MOESM1_ESM.docx]

**Supplementary materials**

**Supplemental Figure 1. Correlation between E/e’ ratio at the 1-year follow-up and LVEF changes (%) from the discharge to the 1-year follow-up.**

**Pearson product-moment correlation coefficient revealed that there was inversely mild correlation between the E/e’ ratio at 1-year follow-up and LVEF changes (%) from the discharge to the 1-year follow-up (*r=-0.25, p=0.02)*.**

**E/e’ = peak velocity of the early wave (E) to early diastole (e’) ratio, LVEF = left ventricular ejection fraction**

**Supplemental Figure 2. Composite outcome after the 1-year follow-up (sensitivity analysis, N = 94)**

The definition of HFrecEF was 1) decreased LVEF <40% at baseline; 2) ≥10% absolute improvement in LVEF; and 3) a second measurement of LVEF >40%.

Cox regression analysis revealed that an E/e’ ratio ≥12.1 at the 1-year follow-up was associated with the composite endpoint after the 1-year follow-up, after adjusting for age and sex (hazard ratio: 4.80, 95% CI: 1.08-21.4).

E/e’ = peak velocity of the early wave (E) to early diastole (e’) ratio; HFrecEF = heart failure with recovered ejection fraction

**Supplemental Figure 3. Composite outcome after the 1-year follow-up according to a cutoff score of LAVI at the 1-year follow-up.**

Kaplan-Meier curve of the composite outcome between the two groups categorized according to the LAVI at the 1-year follow-up, using a cutoff score of 49.4 mL/m2.

LAVI = left atrial volume index

**Supplemental Figure 4. ROC curve of E/e’ ratio at the 1-year follow-up and LVEF changes (%) from the discharge to the 1-year follow-up for composite outcome after the 1-year follow-up in patients with HFrecEF.**

**There was no significant difference between AUC between E/e’ ratio and LVEF changes (E/e’ ratio: 0.70 vs. LVEF changes: 0.61, *p=0.48*).**

**AUC = area under the curve; E/e’ = peak velocity of the early wave (E) to early diastole (e’) ratio; HFrecEF = heart failure with recovered ejection fraction; LVEF = left ventricular ejection fraction; ROC = receiver operating characteristic**
